# Commentary: Toward a more comprehensive autism assessment: the survey of autistic strengths, skills, and interests

**DOI:** 10.3389/fpsyt.2025.1552960

**Published:** 2025-04-29

**Authors:** Grace Joplin Ferreira

**Affiliations:** Faculty of Arts, Department of Languages, University of Helsinki, Helsinki, Finland

**Keywords:** autism (ASC), neurodiversity affirming practice, strengths-based approach, language and communication, autism assessment

The current state of science is constantly changing regarding autism (mis) conceptions (1). Autism research is not only an arena of the status quo of the medical paradigm perpetuated by discourses based on deficit-view accounts of autistic people, but also an arena for bias present in the mindset of researchers; hence, no science is ideology-free (2, 3). Sexism towards women’s agency in the publication of scientific reports on autism, particularly since the development of this term, remains under-investigated. For instance, in 1926, Grunya Efimovna Sukhareva, a Soviet Ukrainian child psychiatrist, differentiated gender differences in autism and separated autistic agency from schizoid traits (4). Despite her contributions, Sukhareva did not receive the same recognition as Kanner or Asperger, and her legacy is still largely ignored (5), further suggesting that science free from ideology does not exist.

The call for shifting the paradigm from a purely medical deficit-based approach to the neurodiversity paradigm which treats differences with dignity and respect is active (6). Shifting the paradigm may lead to investigating what autistics can do instead of what they cannot do (7). Some researchers are responding to this call, like Woods and Estes (8), by rethinking autism in terms of strengths, without necessarily ignoring areas for improvement (9). Considering the imbalance between studies with a deficit-based and strengths-based approach, the SASSI survey by Woods and Estes (8) is indeed a response to the call to action the neurodiversity paradigm has been urging for decades (10–12). Since 2013, Dr. Sara Woods, a neurodivergent clinical psychologist, developed the survey after years of experience and research with autistics at the UW Autism Center in the USA and Dr. Annette Estes, director of the center, revised the SASSI. In both versions of this survey, the questions are based on the strengths of autistics (e.g. honesty and direct communication formulated based on consistent feedback from autistic individuals and their family members, who reported being frequently told they are honest, direct, and sometimes described as having “no filter”; 13–16). This is a significant advancement in autism research, since language in autism is traditionally characterized as an impairment.

Furthermore, SASSI brings a humanistic dialogue centered on the autistic person, not the clinician. The follow-up questions focus purely on the strengths of the individuals such as justice and moral values. The question, “could you think of more examples?” allows extra time for autistics to elaborate their thoughts as they reflect upon their strengths rather than providing most focus on deficits (e.g., “Unusual Eye Contact” item of the ADOS-2; 17).

Woods and Estes’ work demonstrates that researchers with clinical education can reframe their views and methods to embrace autistic experiences. For example, it is documented that autistics have more worries and anxiety than non-autistics about social interaction and how well they will perform it (18). By giving extra time with follow-up questions, the SASSI would be expected to alleviate the stress, anxiety, and worries in face-to-face assessment interactions. It seems that other researchers, especially those from the medical paradigm, are following a similar path as Woods and Estes by not using ableist terminology, such as “high functioning” to refer to adults on the spectrum who do not have intellectual disabilities (19). For instance, the longitudinal study by Clarke and colleagues (2024) carefully crafted the terms more cognitively able (MA) and less cognitively able (LA), thus, avoiding the ableism. This is a small but significant step towards shifting the paradigm, as changing language to refer to autistic people means that these studies in autism do not perpetuate epistemological violence (20–22).

The action for call is also being answered by similar breakthrough projects which, as Woods and Estes, focus on the strengths of neurodivergents. For instance, researchers from the Karolinska Institute in Sweden, with Autistica in the U.K., will release the International Classification of Functioning, Disability and Health (ICF) toolkit for use in education, employment, and healthcare emphasizing strengths and needs of populations with autism and ADHD. This initiative not only focuses on the positive skills of neurodivergent people but also aligns with autistic advocacy for better access to work, rights, and services (14, 23, 24), and supports a shift in autism understanding, as outlined by Huntley et al. (25), with the toolkit based on the ICF framework (26–28). A similar assessment created in Belgium by a father of an autistic child, the Autism Good Feeling Questionnaire (29) is now available in several languages for free.

Woods and Estes hope to further refine the SASSI, including gathering input from autistic adults (the survey is also intended for children). Even though the SASSI is walking towards humanizing autism research in the relationship between clinicians and autistics (30), and it is innovative as it is because it reviews items robustly described as a deficit and disorder, i.e., language and communication (DSM-V) (31), one suggestion is given. According to the authors: “These questions are meant to be asked in interview format as part of a larger clinical interview.” It would be formidable and another advancement for science to test if the SASSI would bring different results when the clinician is autistic versus non-autistic, and if autistic clinicians could provide feedback about their experiences. Doherty et al (32) study declares that when autistic clinicians recognize and disclose themselves as autistics, this has a positive impact on patients’ own recognition and care, therefore legitimizing the autistic agency in the clinical setting. In addition, such a suggestion for the future would not only enhance well-being (33) but also enable more autistic voices in research (34, 35).

## References

[1] Gernsbacher MA Morson EM Grace EJ. Language and Speech in Autism. Annu. Rev. Linguist. (2016) 2:413–25. doi: 10.1146/annurev-linguist-030514-124824 PMC5260808 28127576

[2] Botha M Cage E. Autism research is in crisis”: A mixed method study of researcher’s constructions of autistic people and autism research. Front Psychol. (2022) 13:1050897. doi: 10.3389/fpsyg.2022.1050897 36506950 PMC9730396

[3] Bottema-Beutel K Kapp SK Sasson N Gernsbacher MA Natri H Botha M. Anti-ableism and scientific accuracy in autism research: A false dichotomy. Front Psychiatry. (2023) 14:1244451. doi: 10.3389/fpsyt.2023.1244451 37743979 PMC10514488

[4] Sher DA Gibson JL. Pioneering, prodigious and perspicacious: Grunya Efimovna Sukhareva’s life and contribution to conceptualising autism and chizophrenia. Eur Child Adolesc Psychiatry. (2023) 32:475–90. doi: 10.1007/s00787-021-01875-7 PMC10038965 34562153

[5] Manouilenko I Bejerot S. Sukhareva—Prior to asperger and kanner. Nordic J Psychiatry. (2015) 69:1761–4. doi: 10.3109/08039488.2015.1005022 25826582

[6] Pellicano E den Houting J. Annual Research Review: Shifting from ‘normal science’ to neurodiversity in autism science. J. Child Psychol. Psychiatry Allied Discip. (2022) 63(4):381–96. doi: 10.1111/jcpp.13534 PMC9298391 34730840

[7] Urbanowicz A Nicolaidis C Houting JD Shore SM Gaudion K Girdler S. An expert discussion on strengths-based approaches in autism. Autism Adulthood. (2019) 1:82–9. doi: 10.1089/aut.2019.29002.aju PMC8992818 36601531

[8] Woods SEO Estes A. Toward a more comprehensive autism assessment: The survey of autistic strengths, skills, and interests. Front Psychiatry. (2023) 14:1264516. doi: 10.3389/fpsyt.2023.1264516 37867767 PMC10587489

[9] Kapp SK Gillespie-Lynch K Sherman LE Hutman T. Deficit, dieerence, or both? Autism and neurodiversity. Dev Psychol. (2013) 49:59–71. doi: 10.1037/a0028353 22545843

[10] Davidson J Orsini M. (Eds.). Worlds of autism: Across the spectrum of neurological difference. University of Minnesota Press. (2013).

[11] Dwyer P. The Neurodiversity Approach(es): What Are They and What Do They Mean for Researchers? Hum Dev. (2022) 66(2):73–92. doi: 10.1159/000523723 PMC9261839 36158596

[12] O’Dell L Bertilsdotter Rosqvist H Ortega F Brownlow C Orsini M. Critical autism studies: Exploring epistemic dialogues and intersections, challenging dominant understandings of autism. Disability Soc. (2016) 31:166–79. doi: 10.1080/09687599.2016.1164026

[13] Woods S. Autistic People And The Power Of Positive Onconformity [Online post] (2023). Available online at: https://thinkingautismguide.com/2023/09/autistic-people-and-thepower-of-positive-nonconformity.html (Accessed February 25, 2025).

[14] Cope R Remington A. The strengths and abilities of autistic people in the workplace. Autism Adulthood. (2022) 4:22–31. doi: 10.1089/aut.2021.0037 36605563 PMC8992926

[15] Chevallier C Molesworth C Happé F. Diminished social motivation negatively impacts reputation management: autism spectrum disorders as a case in point. PloS One. (2012) 7:e31107. doi: 10.1371/journal.pone.0031107 22303483 PMC3267764

[16] Kirchner J Ruch W Dziobek I. Brief report: character strengths in adults with autism spectrum disorder without intellectual impairment. J Autism Dev Disord. (2016) 46:3330–7. doi: 10.1007/s10803-016-2865-7 27457365

[17] Lord C Luyster RJ Gotham K Guthrie W. Autism diagnostic observation schedule, second edition (ADOS-2) manual (Part II): toddler module. Torrance, CA: Western Psychological Services (2012).

[18] Black MH Greenwood DL Hwa JCC Pivac J Tang J Clarke PJF. What are you worried about? Content and extent of worry in autistic adults. J Autism Dev Disord. (2024) 54:2040–54. doi: 10.1007/s10803-023-05963-2 PMC10052250 36988767

[19] Bottema-Beutel K Kapp SK Lester JN Sasson NJ Hand BN. Avoiding Ableist Language: Suggestions for Autism Researchers. Autism in Adulthood: Challenges and Management (2021) 3(1):18–29. doi: 10.1089/aut.2020.0014 PMC8992888 36601265

[20] Teo T. What is epistemological violence in the empirical social sciences?: what is epistemological violence in the empirical social sciences? Soc Pers Psychol Compass. (2010) 4:295–303. doi: 10.1111/j.1751-9004.2010.00265.x

[21] Kourti M. A critical realist approach on autism: ontological and epistemological implications for knowledge production in autism research. Front Psychol. (2021) 12:713423. doi: 10.3389/fpsyg.2021.713423 35002826 PMC8732992

[22] Chapman R Carel H H. Neurodiversity, epistemic injustice, and the good human life. J. Soc. Philos. (2022). Advance online publication. doi: 10.1111/josp.12456

[23] Pukki H Bettin J Outlaw AG Hennessy J Brook K Dekker M. Autistic perspectives on the future of clinical autism research. Autism Adulthood. (2022) 4:93–101. doi: 10.1089/aut.2022.0017 36601072 PMC9242721

[24] Kapp SK. Social support, well-being, and quality of life among individuals on the autism spectrum. Pediatrics. (2018) 141:S362–8. doi: 10.1542/peds.2016-4300N 29610418

[25] Huntley M Lee B Falkmer J Bolte P Heasman S Thompson. Action briefing: Strengths-based approaches (2019). Available online at: https://www.autistica.org.uk/downloads/files/FINAL-Strengths-Based-ApproachesActionBriefing.pdf (Accessed February 25, 2025).

[26] Black MH Remnélius KL Alehagen L Bourgeron T Bölte S. From symptomatology to functioning—Applying the ICF to autism measures to facilitate neurodiversity-aeirmative data harmonization. J Autism Dev Disord. (2025) 55(1):114–29. doi: 10.1007/s10803-023-06204-2 PMC11802640 38079032

[27] Alehagen L Hasslinger J Wessman E Black M Lundin Remnélius K Helander J. Operationalizing the ICF Core Sets for Autism and ADHD: A Multiple-Methods Feasibility Study. J Autism Dev Disord. (2025). doi: 10.1007/s10803-024-06717-4 PMC13222184 39883295

[28] Bolte S Alehagen L Black MH Hasslinger J Wessman E Lundin Remnélius K. The Gestalt of functioning in autism revisited: First revision of the International Classification of Functioning, Disability and Health Core Sets. Autism. (2024) 28(9):2394–411. doi: 10.1177/13623613241228896 PMC11402269 38351521

[29] Vermeulen P. Autism good feeling questionnaire (2014). Available online at: https://petervermeulen.be/autism-good-feeling-questionnaire/ (Accessed February 25, 2025).

[30] Whitehead PM Purvis K. Humanizing autism research and treatment: Facilitating individuation through person-centered therapy. Humanistic Psychol. (2023) 51:71–86. doi: 10.1037/hum0000249

[31] American Psychiatric Association, DSM-5 Task Force. Diagnostic and statistical manual of mental disorders: DSM-5™ (5th ed.). American Psychiatric Publishing, Inc. (2013). doi: 10.1176/appi.books.9780890425596

[32] Doherty M Chown N Martin N Shaw SCK. Autistic psychiatrists’ experiences of recognising themselves and others as autistic: A qualitative study. BJPsych Open. (2024) 10:e183. doi: 10.1192/bjo.2024.756 39474853 PMC11698211

[33] Taylor EC Livingston LA Clutterbuck RA Callan MJ Shah P. Psychological strengths and well-being: Strengths use predicts quality of life, well-being and mental health in autism. Autism. (2023) 27(6):1826–39. doi: 10.1177/13623613221146440 PMC10375006 36639858

[34] Keating CT. Participatory autism research: how consultation benefits everyone. Front Psychol. (2021) 12:713982. doi: 10.3389/fpsyg.2021.713982 34504463 PMC8421540

[35] Nicolaidis C Raymaker D Kapp SK Baggs A Ashkenazy E McDonald K. The AASPIRE practice-based guidelines for the inclusion of autistic adults in research as co-researchers and study participants. Autism. (2019) 23:2007–19. doi: 10.1177/1362361319830523 PMC6776684 30939892

